# Preoperative psychological factors influence analgesic consumption and self-reported pain intensity following breast cancer surgery

**DOI:** 10.1186/s12871-024-02622-6

**Published:** 2024-07-16

**Authors:** Khaled Masaud, Audrey Dunn Galvin, Gillian De Loughry, Aisling O. Meachair, Sarah Galea, George Shorten

**Affiliations:** 1 Department of Anaesthesia, Cork University Hospital, Cork, Ireland; 2 Department of Anaesthesia and Intensive Care Medicine, University College of Cork, Cork, Ireland; 3 Department of Anaesthesia, School of Applied Psychology, University College Cork Ireland, University College Cork, Cork, Ireland

**Keywords:** Breast cancer, Preoperative psychological parameters, Anxiety, Postoperative pain

## Abstract

**Background:**

Psychological factors such as anxiety and mood appear to influence acute postoperative pain; however, there is conflicting evidence on the relationship between preoperative psychological parameters and the severity of postoperative pain. In the context of the stressful setting of initial surgery for breast cancer, we conducted a prospective observational study of patients who were scheduled to undergo initial breast cancer surgery.

**Methods:**

The objectives were to examine the potential associations between predefined preoperative psychological parameters and (i) Self-reported pain scores at discharge from the postoperative acute care unit, (ii) Cumulative perioperative opioid consumption at four hours postoperatively and (iii) Self-reported pain as measured during the first seven days after surgery. Patients completed the following questionnaires during the three hours prior to surgery: the Spielberger State Trait Anxiety Inventory (STAI State and Trait), the Pain Catastrophizing Scale (PCS), the Cohen Stress Questionnaire (CSQ), the Hospital Anxiety and Depression Scale (HADS A and D), and the short-form McGill Pain Questionnaire. Postoperative pain experience was assessed using patient self-reports of pain (SF Magill Pain questionnaire on discharge from the postanaesthesia care unit and a pain diary for seven days postoperatively) and records of analgesic consumption.

**Results:**

Pre- to postoperative self-reported pain was significantly different with respect to the STAI State, Cohen score and PCS for both low and high values (*p* < 0.001), but only patients categorized as having low STAI Trait, HADS A, and HADS D values achieved significant differences (*p* < 0.001). A significant positive correlation was demonstrated between preoperative state anxiety (STAI) and the most severe pain reported during the first seven days postoperatively (*r* = 0.271, *p* = 0.013). Patients who were categorized preoperatively as having a “high value” for each of the psychological parameters studied (HADS A and D, STAI State and Trait and PCS) tended to have greater perioperative opioid consumption (up to four hours postoperatively); this trend was statistically significant for HADS D and HADS A only. Using a linear regression model, state anxiety was found to be a significant predictor of postoperative pain based on self-reports during the first seven postoperative days (standardized β = 0.271, t = 2.286, *p* = 0.025).

**Conclusion:**

Preoperative state anxiety, in particular, is associated with the severity of postoperative pain experienced by women undergoing initial breast cancer surgery. Formal preoperative assessment of anxiety may be warranted in this setting with a view to optimize perioperative analgesia and wellbeing.

**Supplementary Information:**

The online version contains supplementary material available at 10.1186/s12871-024-02622-6.

## Background

Breast cancer is the most common malignancy in women, with a global incidence of 43.1 per 100,000, accounting for 25.1% of all cancers. It is the second most common cause of cancer death after lung cancer, with an age-standardized mortality rate of 12.9 (per 100,000) [1]. In Ireland, more than 3,000 women and approximately 25 men are diagnosed with breast cancer each year [2]. As many as 60% of breast cancer surgery patients experience severe acute postoperative pain, which persists for 6–12 months in approximately 10% of patients [3].

The goal of perioperative care is to provide better quality of care to patients before, during and after an operation. Greater levels of preoperative anxiety may be associated with adverse outcomes [4]. Determining any influence of preoperative psychological factors such as anxiety on postoperative pain and recovery is important because it would inform preoperative and personalized and targeted strategies to optimize patient outcomes.

Psychological factors such as anxiety and mood appear to influence acute postoperative pain [5–7]. For instance, anxiety is associated with a decrease in pain perception thresholds [8]. State anxiety reflects temporary psychological and physiological reactions to adverse situations. Trait anxiety describes individual-specific differences in the tendency to attend to, experience, and report negative emotions such as fear, worry and anxiety across many situations. Trait anxiety is, therefore, relatively stable over time [9]. State and trait anxiety are also strongly correlated with each other, indicating that individuals with greater trait anxiety respond with increased state anxiety during an anxiety-provoking situation, such as surgery [9].

Both state and trait anxiety have been shown to correlate positively with the magnitude of postoperative pain [10], including with self-reported pain scores after dental surgery [11]. Elsewhere, it has been reported that state, but not trait anxiety, is associated with more severe postoperative pain [12]. Overall, the evidence regarding the relationship between preoperative anxiety and the severity of postoperative pain is conflicting (with other investigators identifying no significant relationship between anxiety and postoperative pain) [13]. Heterogeneity in design, measures, and predefined outcomes, along with varying quality in study methods, may account for these inconsistencies (12). A similar lack of consistency of evidence exists with respect to the hypothesized association between preoperative depression and postoperative pain [13].

We carried out a prospective observational study of patients who were scheduled to undergo initial breast cancer surgery. The primary objective was to examine the potential associations between predefined preoperative psychological factors and self-reported pain scores at PACU discharge. The secondary objective was to examine potential associations between predefined preoperative psychological factors and cumulative postoperative opioid dose (expressed in oral morphine equivalents) at four hours after surgery and with measures of self-reported pain during the first seven days after surgery.

## Methods

We obtained study approval from the Clinical Research Ethics Committee of the Cork Teaching Hospitals (Reference Numbers ECM 4 on 12/02/2019 and ECM 3 on 03/12/2019). We obtained individual written informed consent from 90 female patients (aged 18–65 years) who underwent primary breast cancer surgery, namely, wide local excision (WLE), WLE and sentinel lymph node biopsy or mastectomy at Cork University Hospital, Ireland. The number of patients recruited for this study represented a convenience sample based on previously reported work that identified positive associations in similar but not identical settings [14, 15].

**Fig. 1:**
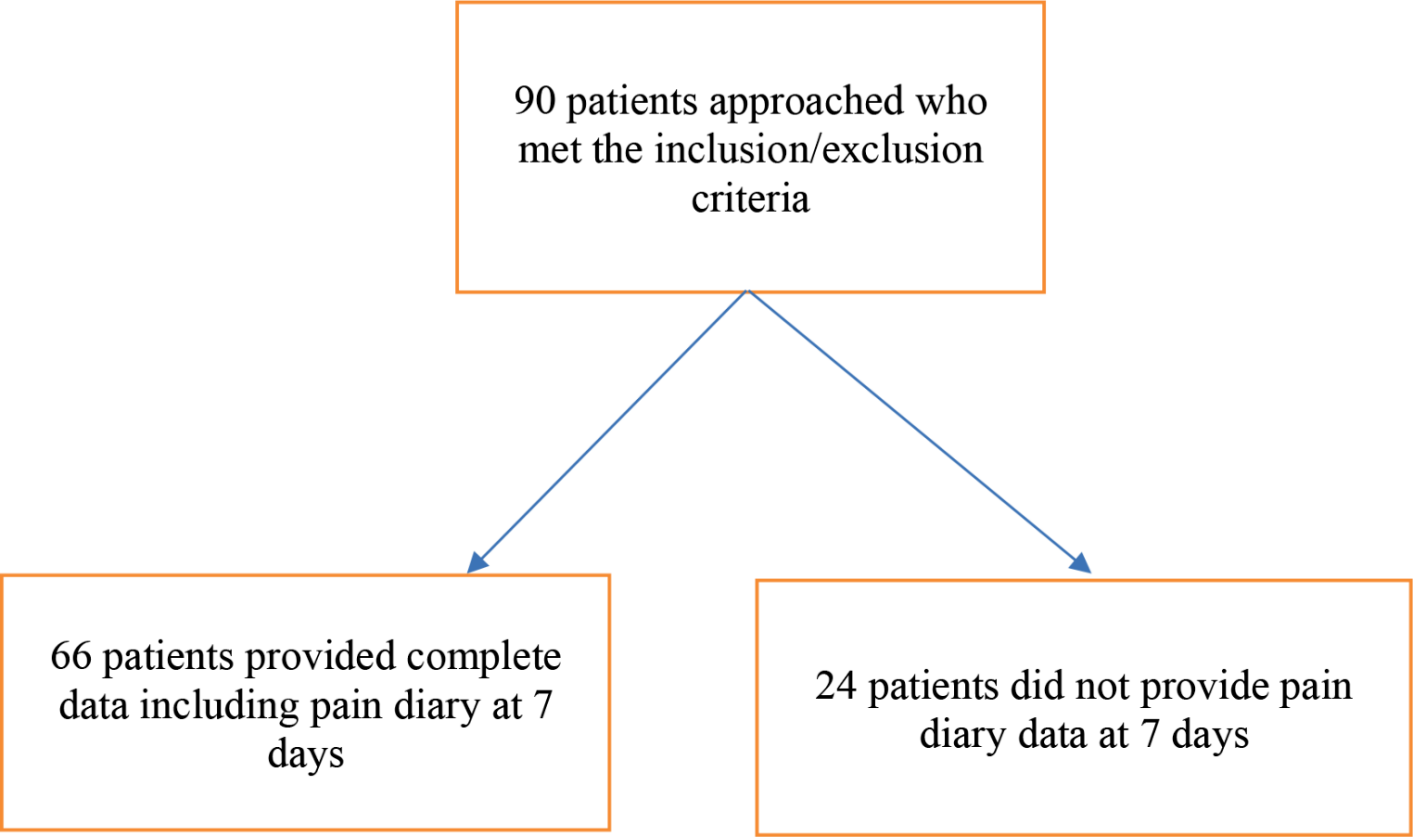
Overview of the number of patients provided data

We examined the patients’ medical records before we approached them, and we only included patients who met the inclusion/exclusion criteria. Of the ninety patients who consented to participate in the study, 66 patients provided complete data, including postoperative pain data, at 7 days, and 24 patients provided preoperative and postoperative PACU data but did not provide 7 days of pain diary data. We did not record the number of patients who declined to participate but met the inclusion/exclusion criteria (Fig. 1).

Exclusion criteria were as follows:

- Preoperative pain of any source requiring analgesic consumption (on more than three occasions) within three months of surgery.
- History of any chronic pain syndrome.
- History of peripheral neuropathy.
- Clinically significant cognitive impairment (Mini Mental State score < 24).
- Any psychological disease condition that required active management.
- Nonfluent in English.
- Obesity (BMI > 35).
- Contraindication to any of the agents administered as part of the standardized perioperative management protocol (see below).

Patients were asked to complete the following questionnaires during the three hours prior to surgery:

- Spielberger’s State Trait Anxiety Inventory (STAI) [16, 17].
- Pain Catastrophizing Score (PCS) [18].
- Cohen Stress Questionnaire (CSQ) [19].
- Hospital Anxiety and Depression Scale (HADS) [17, 20].
- Short-form Pain McGill Questionnaire [21].

All patients underwent standard monitoring applied intraoperatively and received general anaesthesia comprising a standard regimen of 2–3 mg/kg iv propofol, 1 mg/kg iv fentanyl, 0.1–0.15 mg/kg sevoflurane, 4 mg iv ondansetron, 8 mg iv dexamethasone, 1 g iv paracetamol, and 75 mg iv diclofenac. Local anaesthetic infiltration of the surgical site was performed by the surgeon (using approximately 1 mg/kg of 0.5% bupivacaine).

### Preoperative measures

Preoperatively, each patient completed the Spielberger State Trait Anxiety Inventory (STAI), which comprises 20 questions to assess state anxiety and 20 questions to assess trait anxiety. All items are rated on a 4-point scale (e.g., from “Almost Never” to “Almost Always”). Higher scores indicate greater anxiety. For the STAI questionnaire, we used a cut-off threshold score of 40 or greater to indicate high trait anxiety and 45 or greater to indicate high state anxiety (19).

To assess mood preoperatively, we used the Hospital Anxiety and Depression Scale (HADS), which comprises seven items rated on a 4-point severity scale and is scored between zero (no impairment) and three (severe impairment), with a maximum score of 21 for anxiety or depression. A score of 8 or greater is considered to indicate a low mood (17). The PCS Questionnaire comprises 13 statements relating to pain experience, with response options ranging from 0 (not at all) to 4 (all the time). A total score > 5 is considered “high” pain catastrophizing (15).

The Cohen Questionnaire (CSQ) is used to assess the patient’s level of stress. The CSQ comprises 10 items addressing feelings and thoughts during the preceding month. Responses to each question range from 0 = never to 4 = very often. A score > 15 is considered abnormal (16).

The thresholds applied to all the preoperative psychological measures for “low” or “high” values are summarized in Table 1.

**Table 1:** Preoperative psychological parameters in the high and low categories

Psychological parameter	State anxiety	Trait anxiety	CSQ	PCS	HADS A	HADS D
Low	≤40	≤45	≤15	≤5	≤8	≤8
N (%)	48(53)	80(90)	44(49)	45(50)	70(78)	85(94)
High	> 40	> 45	> 15	> 5	> 8	> 8
N (%)	42(47)	9(10)	46(51)	45(50)	20(22)	5(6)

The McGill Pain Questionnaire has three components: Section A for pain character (e.g., burning or throbbing), Section B for pain intensity (e.g., mild, discomforting, distressing, horrible or excruciating) and Section C for pain grade both at rest CR and at movement CM using a verbal rating scale (VRS). The verbal rating scale (VRS) score ranges from 0 (no pain) to 1–3 (moderate pain), 4–5 (moderate pain) and 6–10 (severe pain). All three domains of pain were recorded preoperatively (18).

### Postoperative measures

Postoperative analgesic medication comprised regular paracetamol 1G PO 6 h, regular diclofenac 75 mg PO 12 h, and oxycodone (instant release formulation) 5 mg administered as required at four-hour intervals for breakthrough pain. Patients completed the short-form McGill Pain Questionnaire at postoperative acute care unit (PACU) discharge. The McGill Pain Questionnaire we used here is similar to the McGill Pain Questionnaire used preoperatively. In addition, patients completed a pain diary each day for the subsequent seven days. Cumulative opioid dose: All opioids administered to the patient intraoperatively and postoperatively until four hours after surgery were calculated to represent the total opioid consumption for that period and are expressed as oral morphine equivalents in milligrams.

Pain scores during the seven postoperative days: All patients included in the study were instructed in the use of a pain diary, in which they were asked to record their self-reported pain three times daily for the first seven days postoperatively. A documented verbal rating scale (VRS) ranging from 0 (no pain) to 1–3 (mild pain), 4–5 (moderate pain) and 6–10 (severe pain) was used.

### Statistical analysis

Descriptive statistics were calculated for baseline characteristics. Continuous variables are summarized as the mean (with standard deviation) or median, and categorical variables are summarized as proportions (%). Recommended cut-off points or thresholds for each of the psychological self-report measures were used to categorize high vs. low values (Table 1). To investigate changes in pain from preoperatively to postoperatively, as measured using the Short Form McGill Pain Questionnaire, we used dependent (paired) sample t tests. The independent variable for each test was the psychological self-report measure (categorized as high value or low value group, based on its value relative to the recommended threshold for each). To investigate any differences between preoperative psychological factors and cumulative postoperative opioid dose (expressed in oral morphine equivalents) at four hours after surgery, we used independent sample t tests (categorized based on the value relative to the recommended threshold for each).

No data imputation was performed for missing data. Bonferroni correction was applied to adjust for probability (p) values to guard against the increased risk of a type I error when multiple statistical tests were performed. The p values obtained using two-tailed tests were reported for all the statistical tests; a p value < 0.05 was considered to indicate statistical significance. Statistical analysis was performed using SPSS version 27. Since the data was normally distributed, we used the Pearson Correlation Coefficient Test.

## Results

Our findings revealed a significant positive correlation between preoperative state anxiety (STAI) and the most severe pain reported during the first seven days postoperatively. Patients who were categorized preoperatively as having a “high value” for each of the psychological parameters studied (HADS A and D, STAI State and Trait and PCS) tended to have greater perioperative opioid consumption (up to four hours postoperatively); this trend was statistically significant for HADS D and HADS A only.

**Fig. 2:**
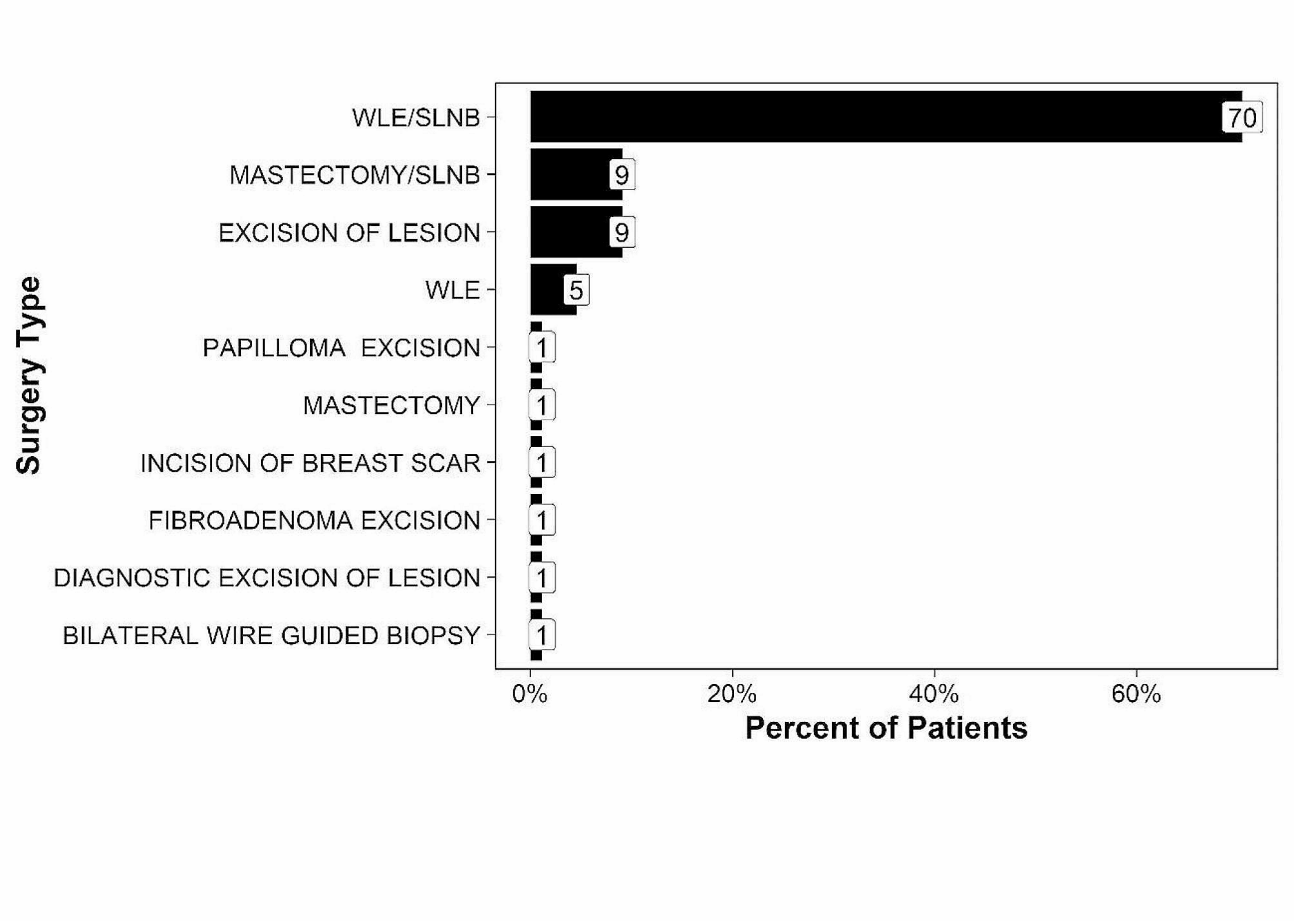
Surgical procedure. The figure displays the percentages of patients who underwent specific surgical procedures. *WLE/SLNB = Wide local excision with sentinel lymph node biopsy

Ninety patients (mean age 54 years; SD 9.6 years) were studied, of whom 88 (70%) underwent wide local excision (WLE) combined with sentinel lymph node biopsy (SLNB) (Fig. 2).

Please refer to the supplementary table for missing data.

Self-reported pain was greater postoperatively than preoperatively according to the McGill A [mean 0.31 (SD 0.58) vs. 1.05 (0.91), respectively], McGill B [mean 0.23 (SD 0.5) vs. mean 1.0 (SD 0.97), respectively], McGill CR [mean 0.49 (SD 1.0)] and McGill CM [mean 0.49 (SD 1.2)]. The mean VRS pain score recorded in the pain diary over the first seven days postoperatively was 1.24 (SD 0.43).

Although the proportion of patients who demonstrated low levels of trait anxiety preoperatively was greater than that who demonstrated high levels (90% vs. 10%, respectively), the equivalent proportions for state anxiety were similar (53% and 47%, respectively).

Preoperatively, all measured scores had a positive and statistically significant correlation, as shown in Table 2.

**Table 2:** Correlation of preoperative psychological parameters

	Cohen *r* (*p*)	STAI State *r* (*p*)	STAI Trait *r* (*p*)	HADS D *r* (*p*)	HADS A *r* (*p*)
PCS	0.372 (< 0.001)	0.358 (0.001)	0.383 (< 0.001)	0.343 (0.001)	0.487 (< 0.001)
Cohen		0.546 (< 0.001)	0.591 (< 0.001)	0.603 (< 0.001)	0.615 (< 0.001)
State anxiety (STAI)			0.482 (< 0.001)	0.399 (< 0.001)	0.570 (< 0.001)
Trait anxiety (STAI)				0.479 (< 0.001)	0.495 (< 0.001)
HADS D					0.735 (< 0.001)

The means and standard deviations (means, SDs) of the preoperative psychological measures (by level) and postoperative McGill Pain Scores are shown in Table 3.

**Table 3:** Preoperative psychological measures (by level) and postoperative McGill Pain scores (means, SDs)

Patient preoperative psychological measure (by level)		Preop McGill A Mean (SD)	Postop McGill A Mean (SD)	Preop McGill B Mean (SD)	Postop McGill B Mean (SD)	Preop McGill CM Mean (SD)	Postop McGill CM Mean (SD)	Preop McGill CR Mean (SD)	Postop McGill CR Mean (SD)
State Anxiety	Low	0.32 (0.594)	1.06 (0.885)	0.21 (0.504)	0.98 (0.838)	0.38 (1.064)	2.38 (2.312)	0.27 (0.818)	2.00 (1.979)
	High	0.31 (0.577)	1.03 (0.957)	0.25 (0.500)	1.00 (1.040)	0.64 (1.313)	2.38 (2.266)	0.57 (1.170)	2.00 (2.103)
Trait Anxiety	Low	0.28 (0.562)	1.08 (0.897)	0.20 (0.493)	1.05 (0.957)	0.40 (1.065)	2.49 (2.336)	0.31 (0.857)	2.09 (2.092)
	High	0.5 (0.756)	0.56 (0.726)	0.38 (0.518)	0.5 (0.527)	1.13 (1.885)	1.5 (1.78)	1 (1.604)	1.2 (1.317)
HADS A	Low	0.33 (0.591)	1.14 (0.926)	0.24 (0.525)	1.09 (0.965)	0.45 (1.091)	2.55 (2.389)	0.35 (0.886)	2.18 (2.112)
	High	0.24 (0.562)	0.82 (0.591)	0.18 (0.393)	0.65 (0.702)	0.69 (1.537)	1.81 (1.759)	0.59 (1.326)	1.18 (1.468)
HADS D	Low	0.32 (0.589)	1.09 (0.923)	0.23 (0.503)	1.01 (0.948)	0.48 (1.17)	2.41 (2.33)	0.38 (0.965)	2.03 (2.050)
	High	0.25 (0.500)	0.75 (0.500)	0.25 (0.500)	0.75 (0.500)	0.75 (1.500)	1.8 (1.3)	0.75 (1.500)	1.00 (1.414)
Cohen	Low	0.29 (0.596)	1.17 (0.961)	0.19 (0.450)	1.07 (0.910)	0.37 (1.070)	2.67 (2.447)	0.28 (0.797)	2.19 (2.107)
	High	0.34 (0.575)	0.98 (0.851)	0.27 (0.549)	0.93 (0.959)	0.63 (1.295)	2.13 (2.102)	0.53 (1.154)	1.75 (1.945)
PCS	Low	0.31 (0.604)	1.07 (0.921)	0.19 (0.450)	1.00 (0.900)	0.37 (1.001)	2.28 (2.119)	0.31 (0.869)	1.86 (1.788)
	High	0.32 (0.574)	1.11 (0.894)	0.29 (0.565)	1.05 (0.985)	0.65 (1.399)	2.65 (2.497)	0.53 (1.133)	2.16 (2.284)

### Primary objectives

Preoperative psychological parameters and pain at postoperative care unit discharge.

Overall, self-reported pain was greater postoperatively than preoperatively according to the McGill A [mean 0.31 (SD 0.58) vs. 1.05 (0.91), respectively], McGill B [mean 0.23 (SD 0.5) vs. mean 1.0 (0.97), respectively], McGill CR [mean 0.49 (SD 1.0)] and McGill CM [mean 0.49 (SD 1.2)]. Patients’ self-reported experience of pain (measured using the McGill SF Pain Questionnaire) across high- and low-value preoperative psychological parameters is summarized in Table 4. For patients categorized preoperatively as either high or low based on state anxiety (STAI), CSQ, and PCS, all measures of pain were significantly greater postoperatively (compared with preoperatively; Table 5). Interestingly, the increase in self-reported pain from pre- to postoperative only achieved statistical significance for patients categorized as low value (but not high value) based on the STAI, HADS A, and HADS D scores (Table 4).

**Table 4:** Comparison of differences in pre- and postoperative McGill Pain scores and preoperative psychological measures

Patient-reported measure		Mean difference McGill A	*P* value	Mean difference McGill B	*P* value	Mean difference McGill CM	*P* value	Mean difference McGill CR	*P* value
**State Anxiety**	Low	0.766	(< 0.001)	0.771	(< 0.001)	2	(< 0.001)	1.729	(< 0.001)
	High	0.75	(< 0.001)	0.778	(< 0.001)	1.8	(< 0.001)	1.73	(< 0.001)
**Trait Anxiety**	Low	0.811	(< 0.001)	0.853	(< 0.001)	2.081	(< 0.001)	1.73	(< 0.001)
	High	0.125	(0.785)	0.125	(0.685)	0.5	(0.649)	0.25	(< 0.001)
**HADS A**	Low	0.803	(< 0.001)	0.851	(< 0.001)	2.104	(< 0.001)	1.833	(< 0.001)
	High	0.588	(0.028)	0.471	(0.016)	1.125	(0.070)	0.588	(0.189)
**HADS D**	Low	0.772	(< 0.001)	0.788	(< 0.001)	1.962	(< 0.001)	1.646	(< 0.001)
	High	0.5	(0.182)	0.500	(0.182)	1	(0.252)	0.250	(0.391)
**Cohen**	Low	0.881	(< 0.001)	0.884	(< 0.001)	2.302	(< 0.001)	1.907	(< 0.001)
	High	0.634	(< 0.001)	0.659	(< 0.001)	1.500	(< 0.001)	1.225	(0.001)
**PCS**	Low	0.762	(< 0.001)	0.814	(< 0.001)	1.907	(< 0.001)	1.548	(< 0.001)
	High	0.789	(< 0.001)	0.763	(< 0.001)	2.000	(< 0.001)	1.632	(< 0.001

*All results remained significant after adjusting for probability (p) values using the Bonferroni correction

**Table 5:** Results for simple and multivariable models predicting the most severe pain score recorded in the pain diary during the first seven days postoperatively

		Unstandardized coefficients		Standardized coefficients*	t	Sig.	95.0% confidence interval for B
Model	Explanatory variables	B	Standard error (S.E)	Beta			Lower bound; upper bound
1 (Simple)	State Anxiety	0.61	0.027	0.27	2.29	0.025	0.01; 0.114
2 (Multivariate)	State Anxiety	0.10	0.031	0.32	2.4	0.018	0.02; 0.14
	Surgery Type	-1.18	0.66	− 0.22	-1.8	0.08	-2.5; 1.3
	Preop McGill A	67	0.95	0.14	0.70	0.49	-1.2; 2.6
	Preop McGill B	1.8	1.5	0.29	1.13	0.26	-1.3; 4.8
	Preop McGill CR	− 0.40	1.44	− 0.14	− 0.28	0.78	-3.3; 2.5
	Preop McGill CM	− 0.77	1.48	− 0.31	− 0.52	0.61	-3.7; 2.2

*Standardized regression coefficients represent the change in the dependent variable in terms of standard deviations for a one-standard-deviation change in the corresponding standardized independent variable. Standardization means that they are “on the same scale” or have the same units, allowing for direct comparison of the magnitude of their effects. Model 1 shows that with every increase of one standard deviation in state anxiety, pain rises by 0.27 standard deviations. Model 2 shows that with every increase of one standard deviation in state anxiety, pain rises by 0.32 standard deviations, holding all other variables constant

### Secondary objectives

Preoperative psychological parameters and cumulative opioid consumption at four hours postoperatively.

The cumulative postoperative analgesic consumption at four hours demonstrated a significant positive correlation with the mean pre- to postoperative difference in the McGill pain score (*r* = 0.249, *p* = 0.018). From an inspection of the means, we identified a trend towards greater postoperative opioid consumption (up to 4 h postoperatively) for patients categorized as having high values for each preoperative psychological parameter (HADS A and D, STAI State and Trait and PCS). This trend was statistically significant for HADS D and HADS A only. Patients categorized as high value (vs. low value) based on HADS D preoperatively had greater opioid consumption during the perioperative period [mean difference 4.5 mg morphine equivalents; (t (40) = 3.45, *p* = 0.001)]. We also observed a significant difference in the mean postoperative cumulative opioid consumption at four hours in the HADS A high value group, with consumption 5.9 mg higher than that in the low value group (i.e., lower anxiety preoperatively) [(t (40) = 3.38, *p* = 0.002)].

### Postoperative pain data for days 1–7

During the first seven days postoperatively, based on self-reports of pain using the VRS recorded in the pain diary, patients experienced mild pain overall (mean VRS 1.74; SD = 2.115); the most severe pain reported during this period was 3.69 (mean; SD = 2.576). A significant positive correlation was observed between preoperative state anxiety (STAI) and the most severe reported pain score on the postoperative pain diary (*r* = 0.271, *p* = 0.013). No significant correlation was identified for the McGill preoperative pain score (in any domain) or the pain score recorded in the pain diary postoperatively. Significant correlations were identified between the most severe pain score on the pain diary and postoperative McGill B (*r* = 0.206, *p* = 0.047), McGill CR (*r* = 0.252, *p* = 0.019), and McGill CM (*r* = 0.209, *p* = 0.046).

Linear regression analysis was therefore conducted to identify predictors of postoperative pain scores during the seven postoperative days as recorded by the pain diary (see Table 5). The first (simple) model included only state anxiety and was statistically significant (R2 = 0.73, F(1, 66) = 5.23, *p* = 0.025). State anxiety significantly predicted postoperative pain (standardized β = 0.271, t = 2.286, *p* = 0.025). A second model controlling for potential confounders, including type of surgery and the McGill SF questionnaire domain, was used. State anxiety (standardized β = 0.336, t = 2.504, *p* = 0.015) remained significant, holding all other variables constant.

## Discussion

Overall, our findings indicate a significant positive correlation between state anxiety (STAI) experienced prior to initial breast cancer surgery and the most severe pain reported during the first seven days postoperatively. A trend was identified for patients categorized as having high values across all the psychological parameters studied (HADS A and D, STAI State and Trait and PCS) to have greater perioperative opioid consumption (up to four hours postoperatively). However, this trend was statistically significant for HADS D and HADS A only. Using a linear regression model, state anxiety was found to be a significant predictor of postoperative pain based on self-reports during the first seven postoperative days (standardized β = 0.271, t = 2.286, *p* = 0.025). A second multivariate model controlling for potential confounders, including type of surgery and all the McGill SF questionnaire domains, was used. According to this model, state anxiety (standardized β = 0.336, t = 2.504, *p* = 0.015) remained significant, while all other variables were not significant. Patients who were more stressed preoperatively had lower pain scores postoperatively, and one possible explanation is that preoperative stress or anxiety might be associated with a greater sense of relief at the conclusion of surgery, and this relief might manifest as less self-reported pain due to a lesser emotional component in the pain experience. Additionally, although a diagnosis has not yet been made here, there is an increased sense of certainty and feeling that the surgery and recovery are actively mitigating the problem.

Given the prospect of surgery, the relatively recent diagnosis of a condition or lesion that requires surgery and the uncertainty relating to prognosis (including the possibility of a negative or even fatal prognosis), patients are exposed to markedly stressful circumstances prior to initial breast cancer surgery. Our findings support the hypothesis that anxiety, which occurs as a result of stressful events or circumstances, tends to be more strongly associated with severe pain. This finding is consistent with the findings of previous investigations, including that of Mimic et al. [6], who demonstrated that following open nephrectomy, anxiety was the most important determinant of pain severity in the early postoperative period. Furthermore, Wang et al. [11] used the Chinese Inventory for Dental Anxiety and Fear (C-IDAF)-4 C to measure anxiety and demonstrated that preoperative dental anxiety and fear, together with surgical time, are associated with postoperative pain following dental surgery and that preoperative anxiety is significantly associated with greater postoperative pain scores. A systematic review by Ip et al. [22] revealed that preoperative pain, anxiety, age and type of surgery were significant predictors of postoperative pain (and that type of surgery, age, and psychological distress were significant predictors of analgesic consumption). Our findings are also consistent with those of a qualitative review by Ip et al. [22], in which all fifteen studies demonstrated a positive correlation between anxiety (state, trait or fear of postoperative pain) and pain intensity. In our study, the influence of state anxiety on pain experience also appeared to extend into the first seven postoperative days and manifested as increased analgesic consumption in the perioperative period (at least until four hours postoperatively). This latter finding is significant because analgesic consumption is not invariably associated with greater degrees of self-reported pain intensity. Pain experience and pain-related behaviours may be complex and poorly understood. For instance, a 2018 study demonstrated that patients with higher catastrophizing scores were more likely to have higher maximum pain scores; however, patients with higher preoperative depression scores were more likely to report a poorer quality of recovery [23].

Our findings indicate that in certain “high stress” circumstances, proactive management of preoperative anxiety may be warranted. A review by Tomaszek and colleagues in 2018 on children and adolescents scheduled to undergo thoracic surgery demonstrated that support from a psychologist offered prior to thoracic surgery can decrease the level of postoperative anxiety [24]. Furthermore, a high level of pre- and postoperative state anxiety may negatively affect patients’ satisfaction with postsurgical analgesia [25]. Formal evaluation of a patient’s anxiety or mood preoperatively may identify those who are at risk of experiencing more severe pain so that appropriate support may be put in place preoperatively. Several investigators [26, 27] have emphasized the importance of preoperative assessment in the early detection of patients with high levels of anxiety so that anxiety can be addressed prior to surgery, for example, through the use of cognitive behavioural therapy [28].

The strengths of this study include the uniformity of the protocol in that it was carried out at a single centre, the anaesthetic technique was rigorously standardized, questionnaires (and pain diaries) were administered by a small number of trained investigators, and the patients were all female and underwent (broadly) similar surgeries with similar diagnostic methods.

However, our study has certain limitations, including the relatively small number of parameters measured, and interpretations regarding secondary outcomes should be made with caution, as the study power was based on differences in 4-hour analgesic consumption. Other factors that need to be considered as potential determinants or predictors of postoperative pain include genotype, age, sex, education, socioeconomic status, past experience with surgery, the precise nature of the proposed surgery, and current health status [28].

Although post hoc analysis showed that the sample size was sufficient for the analyses performed, we did not calculate a sample size prior to recruitment. We noted that the increase in self-reported pain from pre- to postoperative only achieved statistical significance for patients categorized as low value (but not high value) based on STAI Trait, HADS A, and HADS D. This observation may be due to wide interindividual variation in both pain and psychological parameters. Interpretation of these apparently surprising findings should also take into account that baseline values of a continuous variable are negatively correlated with change over time [29]. One important limitation of our study is that we did not evaluate or measure the psychological parameters postoperatively or serially. This means that the anxiety or mood status of the patients studied at the time that postoperative pain measures were elicited or analgesics were consumed is not known. We justified this design (and included predictive modelling) on the basis that measures available to the responsible physician preoperatively were likely to be most valuable in planning an effective analgesic regimen.

A minority (10%) of the patients studied reported high scores for trait anxiety (STAI), which may have skewed the results. Whether the outcome is change score or post score, ideally the baseline should be adjusted for by using analysis of covariance (ANCOVA). However, our sample size was too small to use an ANCOVA, and we therefore accept that our findings have a greater chance of being biased in those with low postoperative scores [29]. In some cases, the use of a change score as the outcome measure is suggested, but we note that this does not address the problem of regression to the mean, nor does it take into account the baseline imbalance [30]. The small sample size makes the significance of the findings less definitive and this study must be used as a baseline for further research and also all significant findings in this study must be interpreted with caution due to the very low numbers in some of the groups.A randomized controlled design should be used with a larger sample size. Our study examined only breast cancer surgery, and all patients were females; therefore, more studies should include a mixture of surgeries and both sexes to determine whether our findings are applicable more broadly.

### Electronic supplementary material

Below is the link to the electronic supplementary material.

Supplementary Material 1

## Data Availability

The data generated and/or analysed during the current study are available from the corresponding author upon reasonable request.

## Abbreviations

STAI State and Trait: Spielberger’s State Trait Anxiety Inventory
PCS: The Pain Catastrophizing Scale
CSQ: The Cohen Stress Questionnaire
HADS A and D: The Hospital Anxiety and Depression Scale
SF Magill Pain Questionnaire: The Short-form McGill Pain Questionnaire
PACU: Postoperative Acute Care Unit
WLE: Wide Local Excision
WLE/SLNB: Wide Local Excision with Sentinel Lymph Node Biopsy
