# Supplementary material for: Preoperative psychological factors influence analgesic consumption and self-reported pain intensity following breast cancer surgery
Source: BMC Anesthesiol. 2024 Jul 16;24:239. doi: 10.1186/s12871-024-02622-6 (PMC11250972; doi:10.1186/s12871-024-02622-6)
Supplement: Supplementary file 1 — Supplementary Material 1 [file 12871_2024_2622_MOESM1_ESM.docx]

**Supplementary Table**

Each value indicates the number of patients for whom data was missing or unavailable either before or after the operation, while percentages represent the proportion of patients with at least one missing data point as a fraction of the total sample (n=90), S1 Table .

S1 Table. Missing data recorded for the final analysis sample (n 90)

| **Variable** | Pre-operative  n (%) | Post-operative  n (%) |
| --- | --- | --- |
| Surgery Type | 0(0) |  |
| State Anxiety | 1(1) |  |
| Trait Anxiety | 2 (2) |  |
| McGill A | 7 (8) | 5 (6) |
| McGill B | 6 (7) | 4 (4) |
| McGill CR | 7 (8) | 4 (4) |
| McGill CM | 6 (7) | 5 (6) |
| Total Opioid Consumption (Oral Morphine equivalent) |  | 16 (18) |
